# Global Spread of Carbapenemase-producing *Enterobacteriaceae*

**DOI:** 10.3201/eid1710.110655

**Published:** 2011-10

**Authors:** Patrice Nordmann, Thierry Naas, Laurent Poirel

**Affiliations:** Author affiliation: Bicêtre Hospital, Le Kremlin-Bicêtre, France

**Keywords:** antimicrobial resistance, bacteria, carbapenems, carbapenemase, Enterobacteriaceae, metallo-β-lactamase, oxacillinase-48, New Delhi metallo-β-lactamase-1, Verona integron–encoded metallo-β-lactamase, IMP, Klebsiella pneumonia carbapenemase, perspective

## Abstract

Carbapenemases increasingly have been reported in *Enterobacteriaceae* in the past 10 years. *Klebsiella pneumoniae* carbapenemases have been reported in the United States and then worldwide, with a marked endemicity at least in the United States and Greece. Metallo-enzymes (Verona integron–encoded metallo-β-lactamase, IMP) also have been reported worldwide, with a higher prevalence in southern Europe and Asia. Carbapenemases of the oxacillinase-48 type have been identified mostly in Mediterranean and European countries and in India. Recent identification of New Delhi metallo-β-lactamase-1 producers, originally in the United Kingdom, India, and Pakistan and now worldwide, is worrisome. Detection of infected patients and carriers with carbapenemase producers is necessary for prevention of their spread. Identification of the carbapenemase genes relies mostly on molecular techniques, whereas detection of carriers is possible by using screening culture media. This strategy may help prevent development of nosocomial outbreaks caused by carbapenemase producers, particularly *K. pneumoniae*.

## Abstract 2

These resistance traits have been identified among nosocomial and community-acquired infections.

*Enterobacteriaceae* are inhabitants of the intestinal flora and are among the most common human pathogens, causing infections such as cystitis and pyelonephritis with fever, septicemia, pneumonia, peritonitis, meningitis, and device-associated infections. *Enterobacteriaceae* are the source of community- and hospital-acquired infections. They have the propensity to spread easily between humans (hand carriage, contaminated food and water) and to acquire genetic material through horizontal gene transfer, mediated mostly by plasmids and transposons.

Since 2000, spread of community-acquired enterobacterial isolates (*Escherichia coli*) that produce extended-spectrum β-lactamases (ESBLs) capable of hydrolyzing almost all cephalosporins except carbapenems has been reported worldwide (*1*). It is therefore mandatory to maintain the clinical efficacy of carbapenems (imipenem, ertapenem, meropenem, doripenem), which have become antimicrobial drugs of last resort. These agents are crucial for preventing and treating life-threatening nosocomial infections, which are often associated with techniques developed in modern medicine (transplantation, hospitalization in an intensive care unit, highly technical surgery).

Carbapenem-resistant *Enterobacteriaceae* have been reported worldwide as a consequence largely of acquisition of carbapenemase genes (*2*). The first carbapenemase producer in *Enterobacteriaceae* (NmcA) was identified in 1993 (*3*). Since then, a large variety of carbapenemases has been identified in *Enterobacteriaceae* belonging to 3 classes of β-lactamases: the Ambler class A, B, and D β-lactamases (*2*). In addition, rare chromosome-encoded cephalosporinases (Ambler class C) produced by *Enterobacteriaceae* may possess slight extended activity toward carbapenems, but their clinical role remains unknown (*2*,*4*).

## Class A Carbapenemases

**Figure 1:**
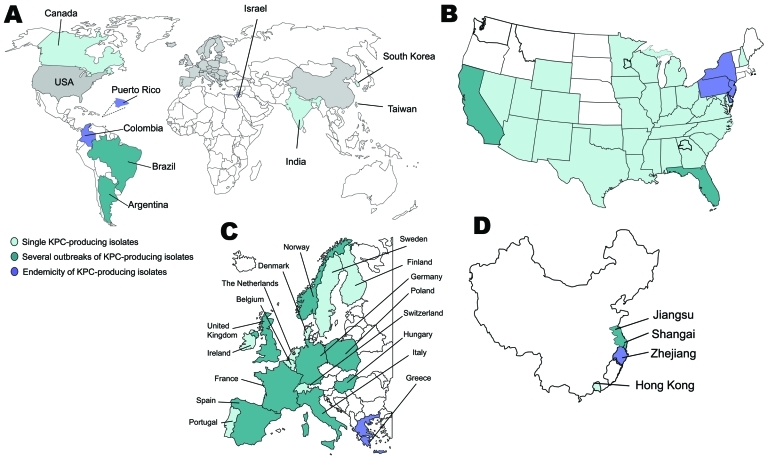
A) Worldwide geographic distribution of *Klebsiella pneumoniae* carbapenemase (KPC) producers. Gray shading indicates regions shown separately: B) distribution in the United States; C) distribution in Europe; D) distribution in China.

A variety of class A carbapenemases have been described; some are chromosome encoded (NmcA, Sme, IMI-1, SFC-1), and others are plasmid encoded (*Klebsiella pneumoniae* carbapenemases [KPC], IMI-2, GES, derivatives), but all effectively hydrolyze carbapenems and are partially inhibited by clavulanic acid (*2*). KPCs are the most clinically common enzymes in this group. The first KPC producer (KPC-2 in *K. pneumoniae*) was identified in 1996 in the eastern United States (*5*).Within a few years, KPC producers had spread globally and have been described across the contiguous United States (still mostly in eastern coast states) and, in particular, in Puerto Rico, Colombia, Greece, Israel, and the People’s Republic of China (*6*,*7*) (Figure 1). Outbreaks of KPC producers also have been reported in many European countries and in South America (*6*,*7*) (Figure 1).

**Figure 2:**
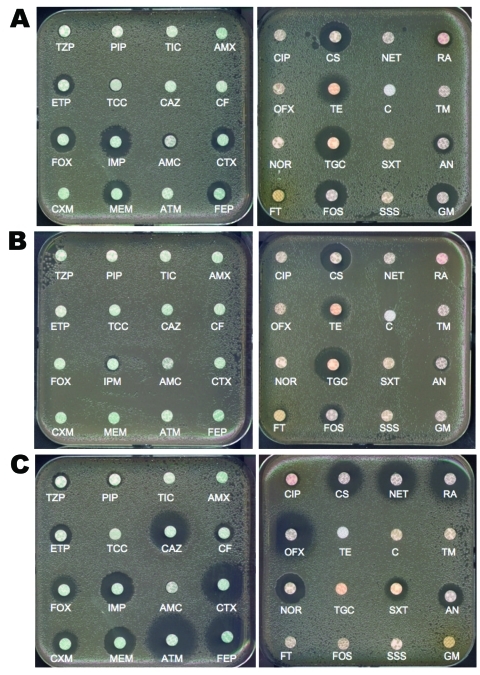
Disk diffusion antibacterial drug susceptibility testing of A) *Klebsiella pneumoniae* carbapenemase-2 (KPC-2)–, B) New Delhi metallo-β-lactamase-1 (NDM-1)–, and C) oxacillinase-48 (OXA-48)–producing *K. pneumoniae* clinical isolates. Clinical isolates producing KPC-2 and OXA-48 do not co-produce other extended-spectrum β-lactamase, but the isolate producing NDM-1 co-produces the extended-spectrum β-lactamase CTX-M-15. Wild-type susceptibility to β-lactams of K. pneumoniae includes resistance to amoxicillin, ticarcillin, and reduced susceptibility to piperacillin and cefalotin (data not shown).TZP, piperacillin/tazobactam; PIP, piperacillin; TIC, ticarcillin; AMX, amoxicillin; ETP, ertapenem; TCC, ticarcillin/clavulanic acid; CAZ, ceftazidime; CF, cefalotin; FOX, cefoxitin; IMP, imipenem; AMC, amoxicillin/clavulanic acid; CTX, cefotaxime; CXM, cefuroxime; MEM, meropenem; ATM, aztreonam; FEP, cefepime; CIP, ciprofloxacin; CS, colistin; NET, netilmicin; RA, rifampin; OFX, ofloxacin; TE, tetracycline; C, chloramphenicol; TM, tobramycin; NOR, norfloxacin; TGC, tigecycline; SXT, sulfamethoxazole/trimethoprim; AN, amikacin; FT, nitrofurantoin; FOS, fosfomycin; SSS, sulfamethoxazole; GM gentamicin.

KPC producers have been reported, mostly from nosocomial *K. pneumoniae* isolates and to a much lesser extent from *E. coli* (especially in Israel) and from other enterobacterial species (*6*). A single *K. pneumoniae* clone (sequence type [ST]-258) was identified extensively worldwide, indicating that it may have contributed to the spread of the *bla*_KPC_ genes (*8*).Within a given geographic location, several KPC clones are disseminating that differ by multilocus sequence type; additional β-lactamase content; and by size, number, and structure of plasmids, but the *bla*_KPC_ genes are associated with a single genetic element (transposon Tn*4401*) (*8*). Although community-acquired KPC producers have been reported, they are rare, with the exception of isolates from Israel a few years ago (*6*).The level of resistance to carbapenems of KPC producers may vary markedly; ertapenem is the carbapenem that has the lowest activity (*5*–*7*), (Table 1). KPC producers are usually multidrug resistant (especially to all β-lactams), and therapeutic options for treating KPC-related infections remain limited (*6*) (Figure 2, panel A). Death rates attributed to infections with KPC producers are high (>50%) (*9**–**11*).

**Table 1:** MIC range of carbapenems for *Enterobacteriaceae* that produce several types of carbapenemases*

Carbapenemase	MIC, mg/L		
	Imipenem	Meropenem	Ertapenem
KPC	0.5–>64	1–>64	0.5–>64
Metallo β-lactamases†	0.5–>64	0.25–>64	0.5–>64
OXA-48 type	1–>64	0.5–>64	0.25–>64

*KPC, *Klebsiella pneumoniae* carbapenemase; OXA-48, oxacillinase-48. †Including New Delhi metallo-β-lactamase-1.

## Class B Metallo-β-Lactamases

**Figure 3:**
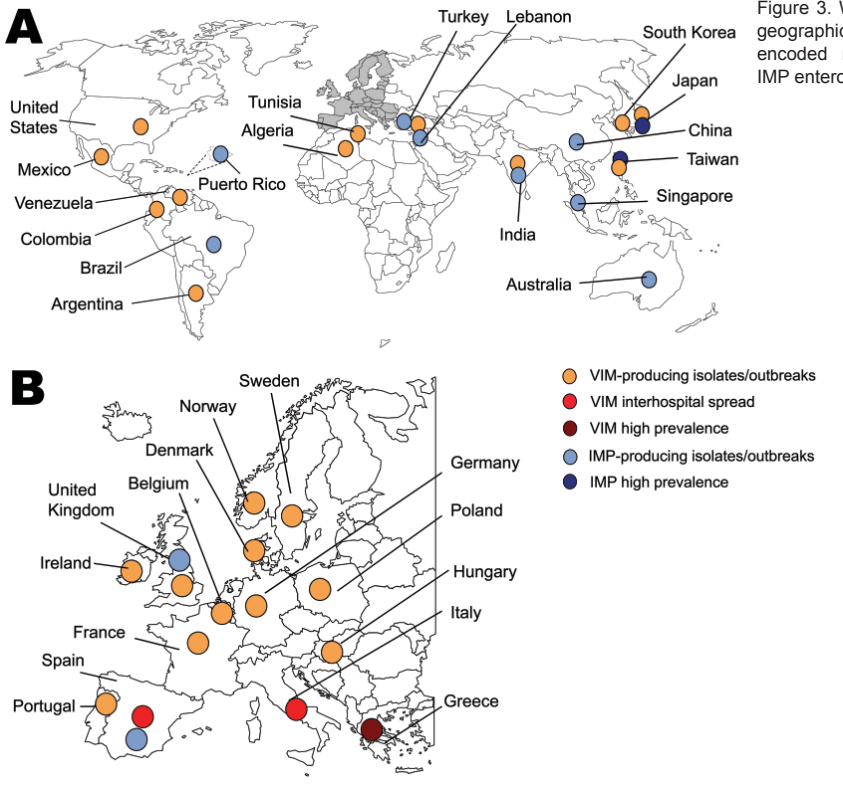
Worldwide (A) and European (B) geographic distribution of Verona integron–encoded metallo-β-lactamase (VIM) and IMP enterobacterial producers.

Class B metallo-β-lactamases (MBLs) are mostly of the Verona integron–encoded metallo-β-lactamase (VIM) and IMP types and, more recently, of the New Delhi metallo-β-lactamase-1 (NDM-1) type (*2**,**12*).The first acquired MBL, IMP-1, was reported in *Serratia marcescens* in Japan in 1991 (*13*). Since then, MBLs have been described worldwide (*2*,*12*) (Figure 3). Endemicity of VIM- and IMP-type enzymes has been reported in Greece, Taiwan, and Japan (*2**,**12*), although outbreaks and single reports of VIM and IMP producers have been reported in many other countries (Figure 3). These enzymes hydrolyze all β-lactams except aztreonam (*12*).Their activity is inhibited by EDTA but not by clavulanic acid (*12*). Most MBL producers are hospital acquired and multidrug-resistant *K. pneumoniae* (*2**,**12*). Resistance levels to carbapenems of MBL producers may vary (Table 1). Death rates associated with MBL producers range from 18% to 67% (*14*).

**Figure 4:**
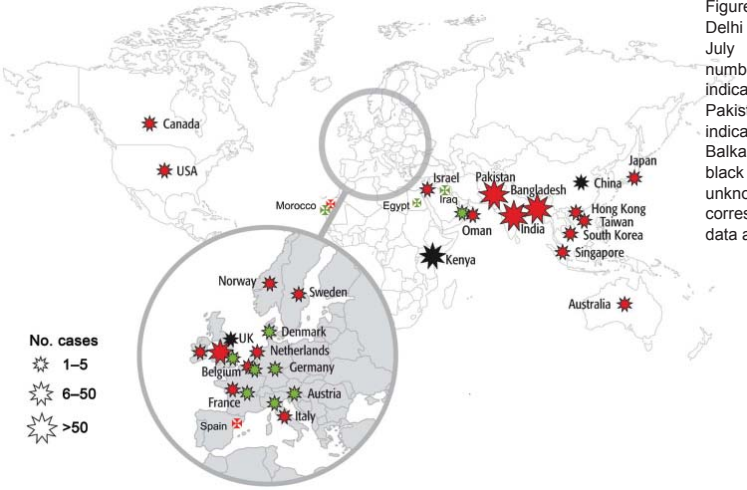
Geographic distribution of New Delhi metallo-β-lactamase-1 producers, July 15, 2011. Star size indicates number of cases reported. Red stars indicate infections traced back to India, Pakistan, or Bangladesh, green stars indicate infections traced back to the Balkan states or the Middle East, and black stars indicate contaminations of unknown origin. (Most of the information corresponds to published data; other data are from P. Nordmann.)

Discovered in 2008 in Sweden from an Indian patient hospitalized previously in New Delhi (*15*), NDM-1–positive *Enterobacteriaceae* are now the focus of worldwide attention (*15**–**17*). Since mid-August 2010, NDM-1 producers have been identified on all continents except in Central and South America with, in most of the cases, a direct link with the Indian subcontinent (*17*) (Figure 4). Few cases have been reported from the United States and Canada (*17*). Recent findings suggest that the Balkan states and the Middle East may act as secondary reservoirs of NDM-1 producers (*17*) (Figure 4).

In contrast to several other carbapenemase genes, the *bla*_NDM-1_ gene is not associated with a single clone but rather with nonclonally related isolates and species (*16**,**17*). It has been identified mostly in *E. coli* and *K. pneumoniae* and to a lesser extent in other enterobacterial species (*16**,**17*). The level of resistance to carbapenems of NDM-1 producers may vary (Table 1). Plasmids carrying the *bla*_NDM-1_ gene are diverse and can harbor a high number of resistance genes associated with other carbapenemase genes (oxacillinase-48 [OXA-48] types, VIM types), plasmid-mediated cephalosporinase genes, ESBL genes, aminoglycoside resistance genes (16S RNA methylases), macrolide resistance genes (esterase), rifampin (rifampin-modifying enzymes) and sulfamethoxazole resistance genes as a source of multidrug resistance and pandrug resistance (*16*,*17*) (Figure 2, panel B). The association of such a high number of resistance genes in single isolates has been rarely observed, even among the other carbapenemase producers. Many NDM-1 producers remain susceptible only to tigecycline, colistin (Figure 2, panel B), and to a lesser extent fosfomycin (*16**,**17*).

Compared with other carbapenemases, NDM-1 has several characteristics that are deeply disconcerting for public health worldwide. These characteristics are 1) occurrence of the *bla*_NDM-1_ gene not in a single species but in many unrelated species and its spread in the environment, at least in the Indian subcontinent (*18*); 2) frequent acquisition by *K. pneumoniae,* a typical nosocomial pathogen, but also by *E. coli* that is by far the main (community-acquired) human pathogen; and 3) size of the reservoir—the Indian subcontinent has >1.4 billion persons. In certain areas in Pakistan, <20% of the population may carry NDM-1 producers (P. Nordmann, unpub. data).

Of particular concern, NDM-1 has been identified in *E. coli* ST-type 131 as a source of community-acquired infection (*19*), an ST type that is known to mobilize efficiently the ESBL CTX-M-15 worldwide (*20*). *E. coli* is the most common cause of diarrhea in children in India. Therefore, this organism may increase the risk of drug-resistant strains being released into the environment and further spread among humans. Accordingly, NDM-1 producers have been recently identified in tap and environmental water in New Delhi, among many unrelated gram-negative species (*18*).

## Class D Enzymes of the OXA-48 Type

**Figure 5:**
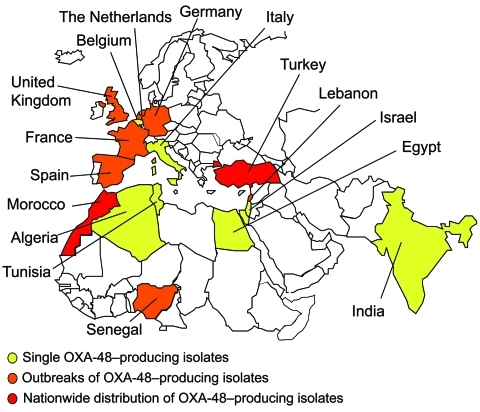
Geographic distribution of oxacillinase-48 (OXA-48) type producers.

The first identified OXA-48 producer was from a *K. pneumoniae* strain isolated in Turkey in 2003 (*21*). Since then, OXA-48 producers have been extensively reported from Turkey as a source of nosocomial outbreaks (*22**–**26*). Their worldwide distribution now includes countries in Europe, in the southern and eastern part of the Mediterranean Sea, and Africa (*21*–*26*) (Figure 5). OXA-48 producers have not been reported from the United States and Canada. A point mutant analog of OXA-48, OXA-181, with similar carbapenemase activity, has been identified in strains from India or of Indian origin (*27**,**28*). There is an increasing trend of identification of OXA-48 producers in countries such as France, Germany, Spain, the Netherlands, and the United Kingdom through transfer of hospitalized patients from disease-endemic areas that are the source of hospital outbreaks (Figure 5).

Several OXA-48–producing clones have been identified, and dissemination of this resistance trait is associated with a 62.5-kb plasmid (previously identified as a plasmid of ≈70 kb) (*22*). OXA-48/OXA-181 are peculiar because they weakly hydrolyze carbapenems and broad-spectrum cephalosporins, such as ceftazidime, and aztreonam (*21*,*27*), (Figure 2, panel C). Their activity is not inhibited by EDTA or clavulanic acid (resistance to amoxicillin/clavulanic acid; Figure 2, panel C). Although reported in various enterobacterial species, OXA-48 producers are mostly identified in *K. pneumoniae* and *E. coli*, and the level of resistance to carbapenems is usually higher when ESBL and permeability defects are associated (*22*–*28*), (Table 1). The OXA-48–type producers are likely the most difficult carbapenemase producers to be identified. Thus, their true prevalence could be underestimated. The attributed mortality rate from infections with OXA-48 producers is unknown.

## Identification of Carbapenemase Producers

The detection of carbapenemase producers in clinical infections is based first on susceptibility testing results obtained by disk diffusion or by automated systems (*29*). The Clinical and Laboratory Standards Institute (CLSI; Wayne, PA, USA) breakpoints of carbapenems have been lowered substantially in 2010 for a better detection of carbapenem-resistant isolates and carbapenemase producers (Table 2). The CLSI breakpoints of carbapenems are now lower than those of the European guidelines (Table 2). Applying the CLSI breakpoints is all that is needed for making treatment decisions according to CLSI recommendations. Special tests for carbapenemase detection are recommended for epidemiology and infection issues.

**Table 2:** Breakpoint values (MIC, mg/L) for carbapenems according to guidelines in Europe (EUCAST) and the United States (CLSI), September 2010*

Carbapenem	EUCAST			CLSI	
	S	R		S	R
Ertapenem	<0.5	>1		<0.25	>1
Imipenem	<2	>8		<1	>4
Meropenem	<2	>8		<1	>4

*EUCAST, European Committee on Antimicrobial Susceptibility Testing (www.eucast.org/clinical_breakpoints); CLSI, Clinical and Laboratory Standards Institute; S, sensitive; R, resistant.

However, low-level resistance and even susceptibility to carbapenems have been observed for producers of any type of carbapenemases (Table 1). We believe, as do others (*30*), that the search for carbapenemase producers should be made for any enterobacterial isolates with decreased susceptibility to carbapenems. Our opinion is based on the paucity of clinical experience for treating infections caused by carbapenemase producers, on the unknown level of carbapenemase production in the site of the infection in vivo, and on the possibility of selecting in vivo for strains with increased levels of resistance to carbapenems and additional mechanisms of carbapenem resistance (carbapenemase, outer-membrane permeability defects).

Specific tests may help identify phenotypically a carbapenemase activity. The modified Hodge test based on in vivo production of carbapenemase has been suggested for detecting carbapenemase producers (*29**,**31**,**32*). However, this test is time consuming and may lack specificity (high-level AmpC producers) and sensitivity (weak detection of NDM producers) (*27**,**29*). This test may be useful for detecting KPC and OXA-48 producers (P. Nordmann, unpub. data). Boronic acid–based inhibition testing is reported to be specific for KPC detection in *K. pneumoniae* when performed with imipenem or meropenem but not with ertapenem if corresponding isolates co-produce a plasmid-mediated AmpC β-lactamase (*29**,**30*). The Etest MBL strip (bioMérieux, Solna, Sweden) is one of the methods advocated for detecting MBL producers on the basis of inhibition of MBL activity by EDTA (*12*). The Etest MBL, using imipenem and imipenem/EDTA, is efficient for detection of MBL producers with high resistance (*12*), but may be deficient for detecting MBL producers with low resistance to imipenem. No inhibition test is available for detection of OXA-48/OXA-181 producers.

Spectrophotometric assay is needed for detecting carbapenemase activity. However, this assay is time consuming, requires specific training, and does not easily discriminate between different types of carbapenemases.

The standard for identification of carbapenemases is based on use of molecular techniques, mostly PCR (*29**,**33*). A list of primers of the most prevalent carbapenemase genes identified in *Enterobacteriaceae* is shown in Table 3 (*34*). Standard conditions may be used for PCR-based detection (*34*). PCR performed on colonies may give results within 4–6 hours with excellent sensibility and specificity. Similarly, other molecular techniques, such as the Check-Points DNA technology, are useful for this purpose (*35*). Sequencing of PCR products may be of interest mostly for epidemiologic purposes. The main disadvantages of molecular-based technologies for detection of carbapenemases are their cost, the requirement of trained personal, and the absence of detection of any novel carbapenemase gene. Thus, there is an urgent need for an inexpensive, rapid, sensitive, and specific test for detection of carbapenemase activity.

**Table 3:** Oligonucleotides used for screening of main carbapenemase genes in Enterobacteriaceae*

Primer	Sequence, 5′ → 3′	Gene	Product size, bp
IMP-F	GGAATAGAGTGGCTTAAYTC	*bla*_IMP_	232
IMP-R	TCGGTTTAAYAAAACAACCACC		
VIM-F	GATGGTGTTTGGTCGCATA	*bla*_VIM_	390
VIM-R	CGAATGCGCAGCACCAG		
OXA-48-F	GCGTGGTTAAGGATGAACAC	*bla*_OXA-48_	438
OXA-48-R	CATCAAGTTCAACCCAACCG		
NDM-F	GGTTTGGCGATCTGGTTTTC	*bla*_NDM_	621
NDM-R	CGGAATGGCTCATCACGATC		
KPC-Fm	CGTCTAGTTCTGCTGTCTTG	*bla*_KPC_	798
KPC-Rm	CTTGTCATCCTTGTTAGGCG		

*A detailed technique for PCR amplification has been reported by Poirel et al. (*34*). VIM, Verona integron–encoded metallo-β-lactamase; OXA, oxacillinase; NDM, New Delhi metallo-β-lactamase-1; KPC, *Klebsiella pneumoniae* carbapenemase.

The prevention of spread of carbapenemase producers relies on early detection of carriers (*29**,**33*). Patients who undergo screening should include patients who were hospitalized while abroad and then transferred to another country, and patients at risk (e.g., patients in intensive care units, transplant patients, immunocompromised patients). Screened patients should be kept in strict isolation before obtaining results of the screening (at least 24–48 hours). Because the reservoir of carbapenemase producers remains the intestinal flora, fecal and rectal swab specimens are adequate for performing this screening. Those specimens may be plated directly on screening media.

There is no universal screening medium able to detect all types of carbapenemase producers with high sensitivity and high specificity, however. Agar plates containing imipenem at a concentration of 1 mg/L have been proposed for screening only KPC producers (*36*). We have demonstrated that a culture medium designed to screen for ESBL producers (ChromID ESBL; bioMérieux, La-Balme-Les-Grotte, France) may be used also for screening carbapenemase producers. Although this medium may lack specificity (co-detection of ESBL producers), its sensitivity is higher than a culture medium designed to screen for carbapenemase producers (CHROMagar KPC; CHROMagar, Paris, France) (*33**,**37*). The main problem remains detection of OXA-48 producers that are susceptible to cephalosporins and have low-level resistance to carbapenems when not co-producing an ESBL (Figure 2, panel C) (*37*). None of these culture media detect those OXA-48 producers (*37*).

After this screening procedure, carbapenemase producers may be identified according to the techniques described above (antibacterial drug susceptibility testing, molecular techniques). Recently, PCR-based techniques performed directly on fecal specimens have been proposed for detection of KPC and NDM-1 producers.

## Conclusions

Carbapenemase producers in *Enterobacteriaceae* are not the source of specific types of clinical infections. The role of these bacteria is related to the difficult-to-treat infections rather than to expression of specific virulence traits.

We believe we are now at the edge of 2 concomitant epidemics of carbapenemase producers worldwide. The first epidemic will be caused mainly by carbapenemase producers in *E. coli* as a source of community-acquired infections. These carbapenemases are thus far primarily of the NDM and of the OXA-48 types. A few published reports of community-acquired infections caused by carbapenemase producers are available, but it is more likely that the numbers of cases in disease-endemic areas are already high. The example of the spread of ESBL producers in the community within the past 10 years shows us that a high rate of carbapenemase producers in *E. coli* may be reached rapidly worldwide. As opposed to a viral epidemic, such as pandemic (H1N1) 2009, the epidemic of carbapenemase producers cannot stop spontaneously. Such community-based outbreaks will be difficult to control. Modulation of the factors that enhance spread of carbapenemase producers in the community is difficult because these factors are multiple and are associated with lack of hygiene, overuse and over-the-counter use of antibacterial drugs, and increased worldwide travel. In addition, many carbapenemase producers carry unrelated drug-resistance determinants. Therefore, selection pressure with structurally unrelated antibacterial drugs (not only β-lactams) may contribute to their spread.

We cannot predict either the speed of diffusion of those carbapenemase producers in the community or their prevalence at a steady state (5%–50%?). The actual prevalence of carbapenemase producers is still unknown because many countries that are likely to be their main reservoirs have not established any search protocol for their detection. The prevalence may substantially differ, depending on the country, as known with the current prevalence rate of ESBL producers in *E. coli*. The prevalence is estimated to be 3%–5% in France and >80% in India (*38*).

The second epidemic will likely be caused mainly by nosocomial carbapenemase producers in *K. pneumoniae* of all types (KPC, IMP, VIM, NDM, and OXA-48). It is likely that in certain countries high rates of different types of carbapenemase producers may already exist, for example, in Greece (VIM and KPC) and in the Indian subcontinent (NDM, KPC, OXA-181). *K. pneumoniae* will play a major role because it has been repeatedly identified to be the most common enterobacterial species for spreading ESBL genes in health care facilities during the past 30 years. It may play the same role for spreading carbapenemase producers in patients with identical risk factors (patients receiving broad-spectrum antibiotherapy, patients in intensive care units, immunocompromised patients, transplant patients, surgical patients). Early identification of carbapenemase producers in clinical infections, at the carriage state, or both, is therefore mandatory to prevent development of those hospital-based outbreaks. We believe we still can efficiently prevent emergence of hospital-based outbreaks of carbapenemase producers. A similar strategy has been implemented in northern European countries for containment of hospital-acquired methicillin-resistant *Staphylococcus aureus*, which has been useful.

The dearth of novel antibacterial drugs in the pipeline means that we must conserve the efficacy of existing antibacterial drugs as much as possible. Carbapenemase producers in *Enterobacteriaceae* are different from other multidrug-resistant bacteria in that they are susceptible to few (if any) antibacterial drugs (*39*).

No vaccines are readily available for preventing infections with carbapenemase producers. This finding is particularly true for *E. coli*, which is part of the human intestinal flora. Therefore, everything must be done to prevent infections as common as pyelonephritis from becoming life threatening because of the lack of any effective treatment.
